# Six-Year Incidence of Visual Impairment in a Multiethnic Asian Population: The Singapore Epidemiology of Eye Diseases Study

**DOI:** 10.1016/j.xops.2023.100392

**Published:** 2023-09-03

**Authors:** Zhi Wei Lim, Miao-Li Chee, Zhi Da Soh, Shivani Majithia, Thakur Sahil, See Teng Tan, Charumathi Sabanayagam, Tien Yin Wong, Ching-Yu Cheng, Yih-Chung Tham

**Affiliations:** 1 Yong Loo Lin School of Medicine, National University of Singapore, Singapore; 2 Singapore Eye Research Institute, Singapore National Eye Centre, Singapore; 3 Eye Academic Clinical Program, Duke-NUS Medical School, Singapore; 4 Tsinghua Medicine, Tsinghua University, Beijing, China; 5 Centre for Innovation and Precision Eye Health & Department of Ophthalmology, Yong Loo Lin School of Medicine, National University of Singapore, Singapore; 6 School of Clinical Medicine, Beijing Tsinghua Changgung Hospital, Beijing, China

**Keywords:** Incidence, Vision impairment, Low vision, Blindness, Asia

## Abstract

**Purpose:**

To examine the 6-year incidence of visual impairment (VI) and identify risk factors associated with VI in a multiethnic Asian population.

**Design:**

Prospective, population-based, cohort study.

**Participants:**

Adults aged ≥ 40 years were recruited from the Singapore Epidemiology of Eye Diseases cohort study at baseline. Eligible subjects were re-examined after 6 years. Subjects included in the final analysis had a mean age of 56.1 ± 8.9 years, and 2801 (50.5%) were female.

**Methods:**

All participants underwent standardized examination and interviewer-administered questionnaire at baseline. Incidences were standardized to the Singapore Population Census 2010. A Poisson binomial regression model was used to evaluate the associations between baseline factors and incident presenting VI.

**Main Outcome Measures:**

Incident presenting VI was assessed at the 6-year follow-up visit. Visual impairment (presenting visual acuity < 20/40), low vision (presenting visual acuity < 20/40 but ≥ 20/200), and blindness (presenting visual acuity < 20/200) were defined based on United States definition.

**Results:**

A total of 5551 subjects (2188 Chinese, 1837 Indians, and 1526 Malays) were evaluated, of whom 514 developed incident presenting VI over 6 years. Malays had a higher incidence of low vision and blindness (13.0%; 0.6%) than Indians (7.0%; 0.1%) and Chinese (7.7%; 0.2%). Among Malay individuals with VI at baseline, 52.8% remained visually impaired after 6 years, which was considerably higher than Chinese (32.4%) and Indians (37.2%). Older age (per decade; relative risk [RR] = 1.59), a history of cardiovascular disease (RR = 1.38), current smoking (RR = 1.31), smaller housing type (1- to 2-room public flat; RR = 2.01), and no formal education (RR = 1.63) at baseline were associated with a higher risk of incident VI (all *P* ≤ 0.027). Older age (> 60 years) contributed the highest population attributable risk to incident VI (27.1%), followed by lower monthly income (Singapore dollar < $2000; 26.4%) and smaller housing type (24.7%). Overall, undercorrected refractive error (49.1%) and cataract (82.6%) were leading causes for low vision and blindness, respectively. This was consistently observed across the 3 ethnicities.

**Conclusions:**

In this multiethnic Asian population, Malays had a higher VI incidence compared to Indians and Chinese. Leading causes of VI are mostly treatable, suggesting that more efforts are needed to further mitigate preventable visual loss.

**Financial Disclosure(s):**

The authors have no proprietary or commercial interest in any materials discussed in this article.

Globally, visual impairment (VI) poses a major public health concern, significantly contributing to the economic and health burden.^1^, ^2^, ^3^ In 2020, it was estimated that 43.3 million people have blindness, while 553 million people have VI.^4^ In view of the aging population, these numbers are projected to increase by approximately 1.5-fold by year 2050.^4^

According to the Global Burden of Disease Study, Asia accounts for > 60% of the total number of VI and blindness cases worldwide.^4^ However, there are limited studies that investigated the incidence of VI and its associated risk factors in Asia.^5^, ^6^, ^7^, ^8^, ^9^, ^10^, ^11^ Previous reports mainly focused on examining the non-Asian populations (Table S1).^12^, ^13^, ^14^, ^15^, ^16^, ^17^, ^18^, ^19^, ^20^ These aspects have not yet been evaluated in the Malay ethnicity, one of Asia’s largest ethnic groups. Furthermore, evaluation of interethnic difference for VI incidence across Asians has not yet been performed. Given the heterogeneity among Asians, this aspect is important and may provide new insights.

Hence, the objective of our study was to examine the 6-year incidence of VI and identify factors associated with VI in a multiethnic Asian population. Findings from this study will contribute toward improving the accuracy of future VI burden projection and associated health care resource allocation. In addition, determining causes and predictors of VI may potentially guide the future formulation of targeted screening and interventions.

## Methods

### Study Populations

We conducted a multiethnic population-based cohort study using subjects recruited from the Singapore Epidemiology of Eye Diseases (SEED) study. At baseline visit, Malay, Indian, and Chinese subjects from SEED were recruited under 3 studies: the Singapore Malay Eye Study (year 2004–2006), the Singapore Indian Eye Study (year 2007–2009), and the Singapore Chinese Eye Study (year 2009–2011). All study procedures adhered to the principles of Declaration of Helsinki,^21^ and ethical approval was obtained from the Singapore Eye Research Institute institutional review board. All subjects provided written informed consent.

In brief, an age-stratified random sampling method was used to select 13 271 subjects (4168 Malays, 4497 Indians, and 4606 Chinese) aged 40–80 years, from which a total of 10 033 subjects (3280 Malays, 3400 Indians, and 3353 Chinese) participated in the study, thereby achieving a response rate of 75.6%. Follow-up visits (SEED-2) were conducted 6 years later (Singapore Malay Eye Study-2, year 2011–2013; Singapore Indian Eye Study-2, year 2013–2015; and Singapore Chinese Eye Study-2, year 2015–2017). The methodology of the baseline and follow-up SEED studies has been previously described in detail.^22^, ^23^, ^24^, ^25^, ^26^

**Figure 1:**
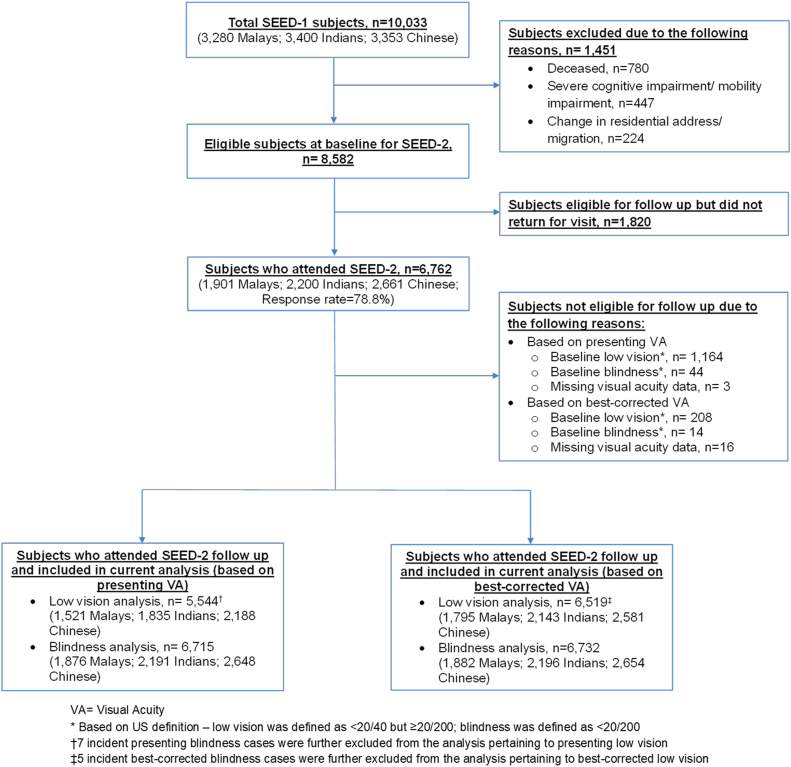
Study population flowchart. SEED = Singapore Epidemiology of Eye Diseases study; US = United States; VA = visual acuity.

At baseline visit, 1451 subjects were excluded due to death (n = 780), severe cognitive impairment/mobility impairment (n = 447), and change in residential address/migration (n = 224). Of the remaining 8582 subjects eligible for SEED-2 follow-up, 1820 subjects were lost to follow-up (response rate: 78.8%). Among the remaining 6762 subjects who attended SEED-2 follow-up, subjects with baseline low vision, baseline blindness, and missing visual acuity (VA) data were excluded accordingly for each analysis. Incident blindness cases were further excluded from the analysis pertaining to incident low vision. Consequently, 5544 subjects and 6715 subjects were included in the final analysis for incident presenting low vision and blindness, respectively. On the other hand, 6519 subjects and 6732 subjects were included in the final analysis for incident best-corrected low vision and blindness, respectively (Fig 1).

### Vision Assessment

Bilateral presenting and best-corrected VA data were collected. Visual acuity was measured at 4m using the logarithm of the minimum angle of resolution number chart (Lighthouse International). In the event where the largest print of a number could not be detected at 4m, the testing distance was sequentially reduced to 3m, 2m, and eventually 1m. If no numbers were read correctly from the logaritham of the minimum angle of resolution number chart at 1m, VA was measured and recorded as counting fingers, hand movement, light perception, or no light perception accordingly. Subjects were instructed to wear their own prescriptive correction, if any, for the measurement of presenting VA, whereas for best-corrected VA, subjects underwent subjective refraction by study investigators.

Visual impairment was defined based on the better-seeing eye according to the United States (US) definition and World Health Organization definition. According to the US definition, VI was defined as VA < 20/40, low vision was defined as VA < 20/40 but ≥ 20/200, and blindness was defined as VA < 20/200. According to the World Health Organization definition, VI was defined as VA < 20/60, low vision was defined as VA < 20/60 but ≥ to 20/400, and blindness was defined as VA < 20/400.

### Other Measurements and Systemic Assessments

An interviewer-administered questionnaire was used to collect data pertaining to subject demographics, medical history, lifestyle risk factors (current smoking status and alcohol intake), and socioeconomic status (education level, type of housing, and monthly income status).

Diabetes mellitus was defined as nonfasting serum glucose ≥ 11.1 mmol/L, glycated hemoglobin ≥ 6.5%, current administration of diabetic medication, or a self-reported history of diabetes mellitus. Hyperlipidemia was defined as either total cholesterol ≥ 6.2 mmol/L or current administration of lipid-lowering medication. Hypertension was defined as systolic blood pressure ≥ 140 mmhg, diastolic blood pressure ≥ 90 mmhg, current administration of antihypertensive medication, or a self-reported history of hypertension. The history of cardiovascular disease (CVD) was defined based on the self-reported history of angina, stroke, or myocardial infarction. Estimated glomerular filtration rate was derived from serum creatinine levels using the Chronic Kidney Disease Epidemiology Collaboration equation.^27^ Chronic kidney disease (CKD) was defined based on the Kidney Disease Outcomes Quality Initiative clinical practice guidelines as estimated glomerular filtration rate < 60 mL/min/1.73 m^2^.^28^, ^29^, ^30^ Body mass index (BMI) was determined using body weight (kilograms) divided by body height (meters) squared. The education level was classified as either receiving formal education or no formal education. Formal education was defined as having primary or higher education. The type of housing was classified as a 1- to 2-room public flat, a 3- to 4-room public flat, or a 5-room public flat and better. The monthly income status was classified as either Singapore dollar (SGD) < $2000 or SGD ≥ $2000.

### Statistical Analysis

All statistical analyses were performed using Stata statistical software (version 13, StataCorp). Age was analyzed as a continuous variable, while gender, ethnicity, diabetes mellitus, hypertension, hyperlipidemia, history of CVD, CKD, BMI, current smoking status, alcohol intake, education level, type of housing, and monthly income status were classified as categorical variables. Independent *t* test and Pearson chi-square test were used for continuous and categorical data, respectively, to compare between subjects with and without incident VI. One-way analysis of variance and chi-square tests were performed for continuous variables and categorical variables, respectively, to compare subject characteristics between different ethnicities.

Visual impairment incidences, based on presenting and best-corrected VA, were evaluated and standardized to Singapore Population Census 2010.^31^ This analysis was stratified by age group (40–49, 50–59, 60–69, and ≥ 70 years), ethnicity (Malay, Indian, and Chinese), and sex. In addition, subjects with baseline VI (i.e., low vision or blindness) were followed up over 6 years to determine the proportion of them who remained visually impaired. The primary causes of both presenting and best-corrected incident VI were also evaluated. A Poisson binomial regression model was used to evaluate the associations between baseline demographic, systemic, and socioeconomic factors with incident presenting VI, best-corrected VI, and the likelihood of remaining visually impaired over 6 years. Population attributable risk (PAR) of factors significantly associated with incident presenting and best-corrected VI was then evaluated, based on the calculated adjusted risk ratio and prevalence of factors.

## Results

A total of 5551 subjects and 6524 subjects were included in the final analysis for incident presenting and best-corrected VI, respectively. Among these subjects, 514 subjects and 222 subjects developed incident presenting and best-corrected VI over 6 years, respectively. In comparison to subjects without incident presenting VI, subjects with incident presenting VI were more likely to be older, to have diabetes mellitus, hypertension, history of CVD, CKD, current smoking, smaller housing, lower monthly income, and no formal education (Table 2, all *P* ≤ 0.028). Similar baseline characteristics were noted for subjects with incident best-corrected VI when compared with subjects without (Table S3, all *P* ≤ 0.029). Comparison of baseline characteristics between ethnicities showed that Malay subjects were more likely to have hypertension, CKD, current smoking, higher BMI, smaller housing, lower monthly income, and no formal education; Indian subjects were more likely to have diabetes mellitus, hyperlipidemia, history of CVD, and alcohol intake; and Chinese subjects were more likely to be older (Table S4, all *P* ≤ 0.001).

**Table 2:** Comparison of Baseline Characteristics Between Subjects With and Without Incident Presenting VI

Baseline Characteristics	Subjects Without Incident Presenting VI∗ (n = 5037)	Subjects With Incident Presenting VI∗ (n = 514)	*P* Value†
Age, years	55.6 (8.6)	61.4 (9.9)	< 0.001
Female gender, n (%)	2557 (50.8)	244 (47.5)	0.155
Ethnicity, n (%)			
Malay	1312 (26.0)	214 (41.6)	< 0.001
Indian	1704 (33.8)	133 (25.9)	
Chinese	2021 (40.1)	167 (32.5)	
Diabetes mellitus, n (%)	1229 (24.4)	179 (34.8)	< 0.001
Hyperlipidemia, n (%)	2144 (43.5)	233 (46.9)	0.152
Hypertension, n (%)	2813 (56.0)	361 (70.4)	< 0.001
History of cardiovascular disease, n (%)	385 (7.6)	70 (13.6)	< 0.001
Chronic kidney disease, n (%)	319 (6.5)	70 (14.3)	< 0.001
Alcohol intake, n (%)	485 (9.6)	33 (6.4)	0.017
Current smoking, n (%)	731 (14.5)	93 (18.1)	0.028
Body mass index, kg/m^2^	25.4 (4.4)	25.4 (4.3)	0.865
Type of housing, n (%)			
1- to 2-room public flat	203 (4.0)	52 (10.2)	< 0.001
3- to 4-room public flat	2915 (58.1)	351 (68.6)	
5-room public flat and better	1903 (37.9)	109 (21.3)	
Monthly income, n (%)			
SGD < $2000	3372 (68.6)	439 (85.7)	< 0.001
SGD ≥ $2000	1544 (31.4)	73 (14.3)	
Education level, n (%)			
No formal education	654 (13.0)	170 (33.2)	< 0.001
Formal education‡	4376 (87.0)	342 (66.8)	

SGD = Singapore dollar; VA = visual acuity; VI = visual impairment.

Data are presented as mean (standard deviation) or number (percentage), where appropriate.

∗ Based on United States definition—VI was defined as VA < 20/40 (i.e., inclusive of low vision and blindness, based on better-seeing eye).

† *P* value was estimated based on chi-square or independent *t* test, where appropriate.

‡ Formal education was defined as having primary or higher education.

Table 5 shows the 6-year incidence of low vision and blindness (US definition). Based on presenting VA, the overall age-standardized incidences for low vision and blindness were 9.4% (95% confidence interval [CI], 8.5%–10.3%) and 0.3% (95% CI, 0.2%–0.5%), respectively. Based on best-corrected VA, the overall age-standardized incidences for low vision and blindness were 3.3% (95% CI, 2.9%–3.8%) and 0.2% (95% CI, 0.1%–0.4%), respectively. The incidence of both presenting and best-corrected VI demonstrated an upward trend with increasing age groups (*P* = 0.001). This was consistently observed across all 3 ethnicities. Notably, for both presenting and best-corrected VI, Malay subjects had the highest incidence compared with Chinese and Indian subjects. Similar trends were observed for World Health Organization-defined low vision and blindness (Table S6). In addition, we also noted generally higher incidences of low vision and blindness in females as compared with males (Table S7).

**Table 5:** Six-year Incidence of Low Vision and Blindness (Based on US Definition, Better-Seeing Eye)

	Vision Status	Age (Year)	Overall		Malay		Indian		Chinese	
			At Risk	Case (%)	At Risk	Case (%)	At Risk	Case (%)	At Risk	Case (%)
Based on presenting VA	Incident low vision∗	40–49	1657	85 (5.1)	487	32 (6.6)	614	32 (5.2)	556	21 (3.8)
		50–59	2087	143 (6.9)	572	54 (9.4)	655	42 (6.4)	860	47 (5.5)
		60–69	1299	164 (12.6)	316	67 (21.2)	426	37 (8.7)	557	60 (10.8)
		70+	501	115 (23.0)	146	56 (38.4)	140	20 (14.3)	215	39 (18.1)
		Total	5544	507 (9.2)	1521	209 (13.7)	1835	131 (7.1)	2188	167 (7.6)
		Age-standardized† (95% CI)	**9.4 (8.5–10.3)**		**13.0 (11.3–15.0)**		**7.0 (5.8–8.4)**		**7.7 (6.5–9.0)**	
	Incident blindness∗	40–49	1805	0 (0.0)	533	0 (0.0)	665	0 (0.00)	607	0 (0.0)
		50–59	2390	3 (0.1)	666	2 (0.3)	759	1 (0.13)	965	0 (0.0)
		60–69	1680	5 (0.3)	419	2 (0.5)	561	2 (0.36)	700	1 (0.1)
		70+	840	15 (1.8)	258	11 (4.3)	206	0 (0.0)	376	4 (1.1)
		Total	6715	23 (0.3)	1876	15 (0.8)	2191	3 (0.1)	2648	5 (0.2)
		Age-standardized† (95% CI)	**0.3 (0.2–0.5)**		**0.6 (0.4–1.1)**		**0.1 (0.02–0.4)**		**0.2 (0.06–0.5)**	
Based on best-corrected VA	Incident low vision∗	40–49	1800	12 (0.7)	530	5 (0.9)	664	5 (0.8)	606	2 (0.3)
		50–59	2370	34 (1.4)	653	19 (2.9)	755	10 (1.3)	962	5 (0.5)
		60–69	1628	75 (4.6)	401	38 (9.5)	541	15 (2.8)	686	22 (3.2)
		70+	721	96 (13.3)	211	46 (21.8)	183	17 (9.3)	327	33 (10.1)
		Total	6519	217 (3.3)	1795	108 (6.0)	2143	47 (2.2)	2581	62 (2.4)
		Age-standardized† (95% CI)	**3.3 (2.9–3.8)**		**5.1 (4.1–6.2)**		**2.1 (1.5–2.9)**		**2.3 (1.8–3.0)**	
	Incident blindness∗	40–49	1809	0 (0.00)	536	0 (0.0)	665	0 (0.0)	608	0 (0.0)
		50–59	2393	1 (0.04)	667	1 (0.2)	760	0 (0.0)	966	0 (0.0)
		60–69	1683	2 (0.1)	418	1 (0.2)	563	1 (0.2)	702	0 (0.0)
		70+	847	11 (1.3)	261	7 (2.7)	208	1 (0.5)	378	3 (0.8)
		Total	6732	14 (0.2)	1882	9 (0.5)	2196	2 (0.09)	2654	3 (0.1)
		Age-standardized† (95% CI)	**0.2 (0.1–0.4)**		**0.4 (0.2–0.8)**		**0.1 (0.01–0.4)**		**0.1 (0.02–0.4)**	

CI = confidence interval; US = United States; VA = visual acuity.

∗ Based on US definition—low vision was defined as VA < 20/40 but ≥ 20/200; blindness was defined as VA < 20/200.

† Incidences were evaluated and standardized to Singapore Population Census 2010.

Table 8 shows the association between baseline factors and incident VI. Following adjustment for age, gender, ethnicity, diabetes mellitus, hyperlipidemia, hypertension, history of CVD, CKD, alcohol intake, current smoking, BMI, type of housing, monthly income, and education level, older age (per decade; relative risk [RR] = 1.59; 95% CI, 1.42–1.77), Malay ethnicity (Chinese as reference: RR = 1.60; 95% CI, 1.28–2.01; Indian as reference: RR = 1.59; 95% CI, 1.27–1.99 [result not shown in table]), history of CVD (RR = 1.38; 95% CI, 1.09–1.76), current smoking (RR = 1.31; 95% CI, 1.03–1.67), smaller housing type (1- to 2-room public flat: RR = 2.01; 95% CI, 1.45–2.79; 3- to 4-room public flat: RR = 1.47; 95% CI, 1.17–1.83), and no formal education (RR = 1.63; 95% CI, 1.34–1.98) were significantly associated with incident presenting VI over 6 years (all *P* ≤ 0.027). Similarly, older age (per decade; RR = 2.30; 95% CI, 1.91–2.78), Malay ethnicity (Chinese as reference: RR = 2.10; 95% CI, 1.44–3.06; Indian as reference: RR = 2.10; 95% CI, 1.44–3.05 [results not shown in table]), history of CVD (RR = 1.47; 95% CI, 1.04–2.10), CKD (RR = 1.56; 95% CI, 1.15–2.12), smaller housing type (1–2 room public flat: RR = 3.63; 95% CI, 2.25–5.87; 3–4 room public flat: RR = 1.70; 95% CI, 1.12–2.57), and no formal education (RR = 1.78; 95% CI, 1.32–2.39) were significantly associated with incident best-corrected VI over 6 years (all *P* ≤ 0.031).

**Table 8:** Association Between Baseline Factors and Incident VI

Baseline Characteristics	Incident Presenting VI∗ (n = 514)		Incident Best-Corrected VI∗ (n = 222)	
	Relative Risk† (95% CI)	*P* Value	Relative Risk† (95% CI)	*P* Value
Age, per decade	1.59 (1.42–1.77)	< 0.001	2.30 (1.91–2.78)	< 0.001
Female gender	0.86 (0.71–1.04)	0.109	0.99 (0.74–1.32)	0.937
Ethnicity				
Chinese	Reference	-	Reference	-
Malay	1.60 (1.28–2.01)	< 0.001	2.10 (1.44–3.06)	< 0.001
Indian	1.05 (0.83–1.32)	0.698	1.01 (0.67–1.51)	0.981
Diabetes mellitus, yes	1.15 (0.95–1.38)	0.148	1.20 (0.90–1.60)	0.222
Hyperlipidemia, yes	0.85 (0.71–1.01)	0.065	0.72 (0.55–0.94)	0.015
Hypertension, yes	1.06 (0.86–1.30)	0.604	1.23 (0.84–1.79)	0.294
History of cardiovascular disease, yes	1.38 (1.09–1.76)	0.009	1.47 (1.04–2.10)	0.031
Chronic kidney disease, yes	0.96 (0.76–1.21)	0.712	1.56 (1.15–2.12)	0.004
Alcohol intake, yes	0.85 (0.60–1.22)	0.380	1.42 (0.82–2.47)	0.210
Current smoking	1.31 (1.03–1.67)	0.027	0.74 (0.46–1.19)	0.216
Body mass index, kg/m^2^	0.99 (0.97–1.01)	0.563	0.95 (0.92–0.99)	0.008
Type of housing				
5-room public flat and better	Reference	-	Reference	-
3- to 4-room public flat	1.47 (1.17–1.83)	0.001	1.70 (1.12–2.57)	0.012
1- to 2-room public flat	2.01 (1.45–2.79)	< 0.001	3.63 (2.25–5.87)	< 0.001
Monthly income, n (%)				
SGD ≥ $2000	Reference	-	Reference	-
SGD < $2000	1.31 (1.00–1.72)	0.050	1.58 (0.85–2.93)	0.144
Education level, n (%)				
Formal education‡	Reference	-	Reference	-
No formal education	1.63 (1.34–1.98)	< 0.001	1.78 (1.32–2.39)	< 0.001

CI = confidence interval; SGD = Singapore dollar; VI = visual impairment.

∗ Based on United States definition—visual impairment was defined as visual acuity < 20/40 (i.e., inclusive of low vision and blindness, based on better-seeing eye).

† Adjusted for age, gender, ethnicity, diabetes mellitus, hyperlipidemia, hypertension, history of cardiovascular disease, chronic kidney disease, alcohol intake, current smoking, body mass index, type of housing, monthly income, and education level.

‡ Formal education was defined as having primary or higher education.

Among the significant risk factors identified (Table 8), older age (> 60 years) contributed the highest PAR to incident presenting VI (27.1%), followed by lower monthly income (SGD < $2000; 26.4%), smaller housing type (24.7%), Malay ethnicity (15.4%), no formal education (14.1%), history of CVD (4.21%), and current smoking (4.19%) (Table S9). Likewise, older age (> 60 years) contributed the highest PAR to incident best-corrected VI (60.4%), followed by smaller housing type (39.5%), no formal education (29.3%), Malay ethnicity (25.3%), CKD (14.3%), and history of CVD (6.11%).

Table 10 shows the primary causes of incident presenting and best-corrected VI over 6 years. Overall, undercorrected refractive error (49.1%; n = 249) and cataract (82.6%; n = 19) were leading causes for presenting low vision and blindness, respectively. Cataract was also the leading cause for best-corrected low vision (71.4%; n = 155) and blindness (64.3%, n = 9). The primary causes of incident presenting and best-corrected VI were similar across all ethnicities (Table S11a, b).

**Table 10:** Primary Causes of Incident Presenting and Best-Corrected VI Over 6 years

Causes of VI∗	Incident Presenting VI∗ (n = 530)		Incident Best-Corrected VI∗ (n = 231)	
	Low Vision∗ (n = 507)	Blindness∗ (n = 23)	Low Vision∗ (n = 217)	Blindness∗ (n = 14)
Undercorrected refractive error	249 (49.1%)	1 (4.3%)	N/A	N/A
Cataract	196 (38.7%)	19 (82.6%)	155 (71.4%)	9 (64.3%)
Diabetic retinopathy	14 (2.8%)	0 (0.0%)	13 (6.0%)	0 (0.0%)
Posterior capsular opacification	13 (2.6%)	1 (4.3%)	8 (3.7%)	2 (14.3%)
Age-related macular degeneration	12 (2.4%)	0 (0.0%)	16 (7.4%)	0 (0.0%)
Maculopathy	5 (1.0%)	0 (0.0%)	10 (4.6%)	0 (0.0%)
Myopia maculopathy	5 (1.0%)	0 (0.0%)	2 (0.9%)	0 (0.0%)
Glaucoma	3 (0.6%)	1 (4.3%)	5 (2.3%)	1 (7.1%)
Amblyopia	2 (0.4%)	0 (0.0%)	2 (0.9%)	0 (0.0%)
Corneal diseases	2 (0.4%)	0 (0.0%)	2 (0.9%)	0 (0.0%)
Retinal vein occlusion	2 (0.4%)	0 (0.0%)	0 (0.0%)	0 (0.0%)
Pterygium	1 (0.2%)	1 (4.3%)	0 (0.0%)	1 (7.1%)
Others	3 (0.6%)	0 (0.0%)	4 (1.8%)	1 (7.1%)

N/A = not applicable; VI = visual impairment.

Data are presented as number (percentage).

∗ Based on United States definition—low vision was defined as visual acuity (VA) < 20/40 but ≥ 20/200; blindness was defined as VA < 20/200, based on better-seeing eye.

Table 12 shows the percentage of subjects with baseline presenting and best-corrected VI who remained visually impaired over 6 years. Overall, 40.1% (n = 485) of subjects with baseline presenting VI remained visually impaired over 6 years. For best-corrected VI, the proportion was 49.1% (n = 109). These percentages were highest in Malay subjects (52.8% for presenting VI; 58.1% for best-corrected VI), followed by Indian subjects (37.2% for presenting VI; 43.9% for best-corrected VI) and Chinese subjects (32.4% for presenting VI; 43.0% for best-corrected VI). In this regard, we evaluated the association between baseline factors and the likelihood of remaining visually impaired over 6 years (Table S13). Following adjustment for the above-mentioned baseline covariates, older age (per decade; RR = 1.17; 95% CI, 1.06–1.29), Malay ethnicity (RR = 1.62; 95% CI, 1.35–1.94), Indian ethnicity (RR = 1.30; 95% CI, 1.06–1.58), monthly income SGD < $2000 (RR = 1.70; 95% CI, 1.16–2.50), and no formal education (RR = 1.23; 95% CI, 1.04–1.45) were significantly associated with the likelihood of remaining visually impaired over 6 years (based on presenting VA; all *P* ≤ 0.018). On the other hand, Malay ethnicity (RR = 1.56; 95% CI, 1.04–2.34) and history of CVD (RR = 1.54; 95% CI, 1.06–2.23) were significantly associated with the likelihood of remaining visually impaired over 6 years (based on best-corrected VA; all *P* ≤ 0.030).

**Table 12:** Percentage of Subjects Who Remained Visually Impaired Over 6 Years∗

Vision Status	Age (Year)	Subjects With Baseline VI∗							
		Overall		Malay		Indian		Chinese	
		n	Remained Visually Impaired Over 6 Years (n [%])	n	Remained Visually Impaired Over 6 Years (n [%])	n	Remained Visually Impaired Over 6 Years (n [%])	n	Remained Visually Impaired Over 6 Years (n [%])
Presenting VI∗ at baseline	40–49	155	42 (27.1)	50	17 (34.0)	53	15 (28.3)	52	10 (19.2)
	50–59	307	108 (35.2)	96	43 (44.8)	104	38 (36.5)	107	27 (25.2)
	60–69	394	140 (35.5)	109	53 (48.6)	138	44 (31.9)	147	43 (29.3)
	70+	352	195 (55.4)	118	84 (71.2)	68	38 (55.9)	166	73 (44.0)
	Total	1208	485 (40.1)	373	197 (52.8)	363	135 (37.2)	472	153 (32.4)
Best-corrected VI∗ at baseline	40–49	11	5 (45.5)	6	4 (66.7)	3	0 (0.0)	2	1 (50.0)
	50–59	23	9 (39.1)	13	7 (53.8)	5	0 (0.0)	5	2 (40.0)
	60–69	61	27 (44.3)	19	9 (47.4)	24	9 (37.5)	18	9 (50.0)
	70+	127	68 (53.5)	48	30 (62.5)	25	16 (64.0)	54	22 (40.7)
	Total	222	109 (49.1)	86	50 (58.1)	57	25 (43.9)	79	34 (43.0)

VI = visual impairment.

∗ Based on United States definition—visual impairment was defined as visual acuity < 20/40, based on better-seeing eye. Include subjects with low vision or blindness at baseline who remained status quo over 6 years and those with low vision at baseline who deteriorated to blindness over 6 years.

## Discussion

In this multiethnic Asian population, we evaluated the incidence and risk factors associated with incident VI over 6 years. Malays consistently demonstrated the highest incidence of VI across all age groups. Among subjects with VI at baseline, > 40% remained visually impaired after 6 years. Importantly, the leading causes of incident VI were undercorrected refractive error and cataract, both of which are readily treatable. To the best of our knowledge, this was the first multiethnic population-based Asian study to examine the incidence of VI. Notably, our multiethnic study cohort provided us with a unique opportunity to evaluate the 3 main ethnicities in Asia, which represent > 70% of Asia’s ethnic composition.^32^ Our study findings are pertinent in further improving the accuracy of future VI burden projection and its associated health care resource allocation.

Compared to studies conducted in non-Asian populations, our incidence (based on US definition) was higher for presenting low vision but generally lower for best-corrected low vision (Table S1). On the other hand, when comparing our annual incidence with other Asian studies, we observed some disparities. The annual incidence of VI among Indians in our study was lower than that reported in the Andhra Pradesh Eye Disease Study. However, for the Chinese population, our VI annual incidence was higher than that in the Beijing Eye Study yet lower than those in both the Liwan Eye Study and the Zhongshan Ophthalmic Center Study. However, it is important to note that these comparisons are somewhat rudimentary, as they are not standardized to common population census data. Furthermore, these studies are inherently different in terms of study methodology and subject population. The evaluation of VI incidence was based on different definitions, and study participants had varying age groups, ethnicity, and socioeconomic backgrounds. Therefore, comparisons between study findings should be interpreted with care.

Consistent with previous population studies,^5^, ^6^, ^7^, ^8^, ^9^, ^10^, ^11^, ^12^, ^13^, ^14^^,^^16^, ^17^, ^18^^,^^20^ we found a significant association between older age and incident VI. This relationship was anticipated as multiple common causes of VI, including age-related macular degeneration and cataracts, are strongly associated with aging. Furthermore, systemic conditions like diabetes and hypertension, which may be complicated by sight-threatening conditions such as diabetic and hypertensive retinopathy, are also associated with aging.^33^, ^34^, ^35^, ^36^ We also observed that Malays were approximately 1.5–2 times as likely to develop incident presenting and best-corrected VI compared to Indians and Chinese, in spite of relatively equal access and subsidies to health care services in Singapore. Despite being one of Asia’s largest ethnic groups, there are no previous cohort studies that evaluated this aspect.

Among the baseline systemic conditions evaluated, history of CVD and CKD demonstrated a significant association with incident best-corrected VI, suggesting a potential link between these systemic conditions and ocular pathologies. In this regard, CKD and CVD share common disease risk factors and pathologic pathways with several ocular diseases.^37^, ^38^, ^39^ Taken together, screening for systemic diseases and administering timely interventions may mitigate against incident VI.

In our study, we also observed that individuals with smaller housing type and no formal education, both being surrogates of lower socioeconomic status, were associated with incident presenting and best-corrected VI. Previous reports indicated that individuals of lower socioeconomic status were observed to be less likely to participate in health screening and more inclined toward alternative medicine.^40^ Specifically, lower levels of educational attainment may directly influence an individual’s health literacy and health-seeking behavior,^41^ placing them at higher risk of ocular diseases and consequently VI.

The leading causes of incident presenting and best-corrected VI were undercorrected refractive error and cataract, respectively, both cumulatively accounting for the majority of the VI cases. Importantly, these 2 conditions are readily treatable, and intervention may vastly improve quality of life.^42^^,^^43^ By simply correcting for refractive error and providing access to cataract surgery, a substantial proportion of low vision in the community may be circumvented.

Remarkably, > 40% of subjects with baseline VI remained visually impaired over 6 years (Table 12). In this regard, we identified several baseline factors associated with these individuals, namely older age, Malay ethnicity, Indian ethnicity, history of CVD, monthly income SGD < $2000, and no formal education (Table S13). This information is pertinent for formulating targeted public health interventional strategies for secondary and tertiary prevention to ameliorate the burden of VI.

The strengths of this study include its large population-based sample, comprising 3 of the largest ethnicities in Asia. In addition, we utilized a robust and standardized methodology which enabled the evaluation of several baseline factors and adjustment for multiple important confounders. The longitudinal cohort study design also provided us insights into the progression of individuals with VI at baseline and the proportion which remained visually impaired since baseline. Furthermore, we performed multifaceted evaluations on causes, predictive risk factors, and PAR among identified factors. Nonetheless, our study has its limitations. First, visual field deficit was not included as a criterion in our definition of VI. This may potentially result in slight underestimation of the incidence of VI due to glaucoma. Second, among 8582 subjects who were eligible at baseline for SEED-2, 1820 subjects did not return for follow-up. Hence, loss to follow-up bias, an intrinsic weakness of every cohort study, cannot be entirely excluded. In this regard, subjects excluded from the final analysis for incident presenting VI were older, more likely to be Indians, Malays, smokers, have diabetes, hypertension, history of CVD, CKD, higher BMI, smaller housing type, lower monthly income, and no formal education (Table S14). Given that several of these baseline characteristics are associated with incident VI, the incidence presented in our study may be marginally underestimated.

In conclusion, in this multiethnic Asian adult population, 9 in 100 developed presenting low vision and 3 in 1000 developed blindness over 6 years. Compared to Indians and Chinese, Malays had the highest incidence of VI. Importantly, refractive error and cataract are the leading causes of VI, and both are readily treatable with cost-effective interventions. Our study findings are pertinent in further improving the accuracy of future burden projection and formulating health care strategies to alleviate the burden of VI.
