# Supplementary material for: Six-Year Incidence of Visual Impairment in a Multiethnic Asian Population: The Singapore Epidemiology of Eye Diseases Study
Source: Ophthalmol Sci. 2023 Sep 3;3(4):100392. doi: 10.1016/j.xops.2023.100392 (PMC10630666; doi:10.1016/j.xops.2023.100392)
Supplement: Supplementary Data [file mmc1.pdf]

Table S1. Previous Studies which Evaluated Incidence of Visual Impairment, Based on US and WHO definition

Country	Study	Author (Year)	Years of follow up	Age Mean (±SD)	Cumulative Incidence Based on US Definition* (%)				Cumulative Incidence Based on WHO definition** (%)			
					Presenting Low Vision	Presenting Blindness	Best-Corrected Low Vision	Best-Corrected Blindness	Presenting Low Vision	Presenting Blindness	Best-Corrected Low Vision	Best-Corrected Blindness
**Asia**												
**Singapore**	SEED	Current Study	6	56.1 (±8.9)	9.2	0.3	3.3	0.2	4.1	0.2	1.3	0.1
**China**	BES[1]	You, 2011	5	55.3 (±10.1)	3.8	-	1.1	0.1	1.7	-	0.5	0.1
	Handan[2]	Hu, 2020	6	51.4 (±11.1)	-	-	-	-	7.4	0.6	1.6	0.1
	Liwan[3]	Wang, 2013	5	63.4 (±9.0)	20.6	1.77	9.85	1.42	12.4	0.33	5.38	0.33
	ZOCS[4]	Han, 2019	6	52.3 (±11.6)	12.2†	0.14†	-	-	8.33†	0†	-	-
**India**	CEDIS[5]	Vijaya, 2014	6	52.8 (±9.7)	-	-	-	-	-	-	-	0.48
	APEDS[6]	Khanna, 2021	15	28.0 (±17.5)	25.5	3.6	12.9	1.8	-	1.3	-	1.3
**Iran**	ShECS[7]	Hashemi, 2016	5	50.9 (±6.2)	-	-	-	-	1.02	0.13	0.11	0.09
**Africa**												
**Kenya**	NEDCS[8]	Bastawrous, 2016	6	62.5 (±9.3)	-	2.59	-	-	-	1.51	-	-
**Americas**												
**Caribbean**	BISED[9]	Hennis, 2009	9	-‡	-	-	6.4	2.1	-	-	4.5	1.0
**North America**	BDES[10]	Klein, 2013	20	75.4 (-)	-	-	-	0.4	-	-	-	-
	LALES[11]	Varma, 2010	4	54.7 (±10.5)	2.9†	0.3†	1.2	0.3	1.8†	0.2†	0.7	0.2
**Europe**												
**Iceland**	Reykjavik[12]	Gunnlaugsdottir, 2010	5	64.7 (±9.7)	-	-	3.49	0.95	-	-	1.07	0.35
**Italy**	Ponza[13]	Nucci, 2005	12	55.5 (±9.4)	-	-	-	-	-	-	3.9	0.7
	Priverno[14]	Cedrone, 2002	7	57.0 (±6.7)	-	-	-	-	-	-	1.3	0.2
**Oceania**												
**Australia**	BMES[15]	Hong, 2013	15	64.3 (±8.6)	-	-	-	0.9	-	-	-	-
	MVIP[16]	Dimitrov, 2003	5	59.0 (±12.0)	3.72	0.43	-	-	1.11	0.28	-	-

SD= Standard Deviation; SEED= Singapore Epidemiology of Eye Diseases; BES= Beijing Eye Study; ZOCS= Zhongshan Ophthalmic Center Study; CEDIS= Chennai Eye Disease Incidence Study; APEDS= Andhra Pradesh Eye Disease Study; ShECS= Shahroud Eye Cohort Study; NEDCS= Nakuru Eye Disease Cohort Study; BISED= Barbados Incidence Study of Eye Diseases; BDES= Beaver Dam Eye Study; LALES= Los Angeles Latino Eye Study; BMES= Blue Mountain Eye Study; MVIP= Melbourne Visual Impairment Project

*US definition based on visual acuity of better-seeing eye – low vision was defined as <20/40 but ≥20/200; blindness was defined as <20/200

**WHO definition based on visual acuity of better-seeing eye – low vision was defined as <20/60 but ≥20/400; blindness was defined as <20/400

†Low vision and blindness was defined using binocular presenting visual acuity

‡Mean (±SD) of age was not included in manuscript; age range of participants at baseline was 40 to 84 years.

1. You QS, Xu L, Yang H, Wang YX, Jonas JB. Five-year incidence of visual impairment and blindness in adult Chinese the Beijing Eye Study. *Ophthalmology*. Jun 2011;118(6):1069-75. doi:10.1016/j.ophtha.2010.09.032
2. Hu A, Gu SZ, Friedman DS, Cao K, Wang N. Six-Year Incidence and Causes of Low Vision and Blindness in a Rural Chinese Adult Population: The Handan Eye Study. *Ophthalmic Epidemiol*. Apr 2021;28(2):160-168. doi:10.1080/09286586.2020.1795886
3. Wang L, Huang W, He M, et al. Causes and Five-Year Incidence of Blindness and Visual Impairment in Urban Southern China: The Liwan Eye Study. *Investigative Ophthalmology & Visual Science*. 2013;54(6):4117-4121. doi:10.1167/iovs.13-11911
4. Han X, Liao C, Liu C, et al. Incidence and correction of vision impairment among elderly population in southern urban China. *Clin Exp Ophthalmol*. May 2019;47(4):439-444. doi:10.1111/ceo.13431
5. Vijaya L, Asokan R, Panday M, et al. Baseline Risk Factors for Incidence of Blindness in a South Indian Population: The Chennai Eye Disease Incidence Study. *Investigative Ophthalmology & Visual Science*. 2014;55(9):5545-5550. doi:10.1167/iovs.14-14614
6. Khanna RC, Marmamula S, Pendri P, et al. Incidence, Incident Causes, and Risk Factors of Visual Impairment and Blindness in a Rural Population in India: 15-Year Follow-up of the Andhra Pradesh Eye Disease Study. American journal of ophthalmology. Mar 2021;223:322-332. doi:10.1016/j.ajo.2020.09.035
7. Hashemi H, Mehravaran S, Emamian MH, Fotouhi A. Five-Year Incidence of Visual Impairment in Middle-Aged Iranians: The Shahroud Eye Cohort Study. *Ophthalmic Epidemiol*. Feb 2017;24(1):11-16. doi:10.1080/09286586.2016.1255342
8. Bastawrous A, Mathenge W, Wing K, et al. Six-Year Incidence of Blindness and Visual Impairment in Kenya: The Nakuru Eye Disease Cohort Study. *Investigative ophthalmology & visual science*. 2016;57(14):5974-5983. doi:10.1167/iovs.16-19835
9. Hennis AJ, Wu SY, Nemesure B, Hyman L, Schachat AP, Leske MC. Nine-year incidence of visual impairment in the Barbados Eye Studies. *Ophthalmology*. Aug 2009;116(8):1461-8. doi:10.1016/j.ophtha.2009.02.017
10. Klein R, Lee KE, Gangnon RE, Klein BE. Incidence of visual impairment over a 20-year period: the Beaver Dam Eye Study. *Ophthalmology*. Jun 2013;120(6):1210-9. doi:10.1016/j.ophtha.2012.11.041
11. Varma R, Chung J, Foong AW, Torres M, Choudhury F, Azen SP. Four-year incidence and progression of visual impairment in Latinos: the Los Angeles Latino Eye Study. *Am J Ophthalmol*. May 2010;149(5):713-27. doi:10.1016/j.ajo.2009.12.011
12. Gunnlaugsdottir E, Arnarsson A, Jonasson F. Five-year incidence of visual impairment and blindness in older Icelanders: the Reykjavik Eye Study. https://doi.org/10.1111/j.1755-3768.2008.01445.x. *Acta Ophthalmologica*. 2010/05/01 2010;88(3):358-366. doi:https://doi.org/10.1111/j.1755-3768.2008.01445.x
13. Nucci C, Cedrone C, Culasso F, et al. Incidence of visual loss in the Ponza Eye Study, Italy. *Eye (Lond)*. Feb 2005;19(2):175-82. doi:10.1038/sj.eye.6701444
14. Cedrone C, Culasso F, Cesareo M, et al. Incidence of blindness and low vision in a sample population: the Priverno Eye Study, Italy. *Ophthalmology*. Mar 2003;110(3):584-8. doi:10.1016/s0161-6420(02)01898-5
15. Hong T, Mitchell P, Rochtchina E, Fong CS, Chia EM, Wang JJ. Long-term changes in visual acuity in an older population over a 15-year period: the Blue Mountains Eye Study. *Ophthalmology*. Oct 2013;120(10):2091-9. doi:10.1016/j.ophtha.2013.03.032
16. Dimitrov PN, Mukesh BN, McCarty CA, Taylor HR. Five-year incidence of bilateral cause-specific visual impairment in the Melbourne Visual Impairment Project. *Invest Ophthalmol Vis Sci*. Dec 2003;44(12):5075-81. doi:10.1167/iovs.02-0457

Table S3. Comparison of Baseline Characteristics between Subjects with and without Incident Best-Corrected Visual Impairment

Baseline characteristics	Subjects without incident best-corrected VI* (n= 6,302)	Subjects with incident best-corrected VI* (n=222)	P-value†
Age, years	56.6 (9.0)	67.4 (9.0)	<0.001
Female Gender, n (%)	3244 (51.5)	118 (53.2)	0.623
Ethnicity, n (%)			
• Malay	1687 (26.8)	113 (50.9)	<0.001
• Indian	2096 (33.3)	47 (21.2)	
• Chinese	2519 (40.0)	62 (27.9)	
Diabetes Mellitus, n (%)	1588 (25.2)	89 (40.1)	<0.001
Hyperlipidaemia, n (%)	2728 (44.3)	100 (47.8)	0.312
Hypertension, n (%)	3631 (57.8)	183 (82.4)	<0.001
History of Cardiovascular Disease, n (%)	514 (8.2)	41 (18.5)	<0.001
Chronic Kidney Disease, n (%)	445 (7.3)	64 (30.9)	<0.001
Alcohol Intake, n (%)	585 (9.3)	13 (5.9)	0.081
Current Smoking, n (%)	927 (14.7)	21 (9.5)	0.029
Body Mass Index, kg/m^2	25.4 (4.5)	24.9 (4.4)	0.113
Type of Housing, n (%)			
• 1-2 room public flat	287 (4.6)	44 (19.9)	<0.001
• 3-4 room public flat	3781 (60.2)	144 (65.2)	
• 5 room public flat and better	2216 (35.3)	33 (14.9)	
Monthly Income, n (%)			
• <SGD $2,000	4418 (71.8)	208 (94.1)	<0.001
• ≥SGD $2,000	1739 (28.2)	13 (5.9)	
Education Level, n (%)			
• No formal education	1059 (16.8)	119 (53.6)	<0.001
• Formal education‡	5232 (83.2)	103 (46.4)	

VI= Visual Impairment, SGD= Singapore Dollars

Data presented as mean (standard deviation) or number (percentage), where appropriate.

*Based on US definition – VI was defined as VA<20/40 (i.e. inclusive of low vision and blindness, based on better-seeing eye).

†P-value was estimated based on chi-squared or independent t-test, where appropriate

‡Formal education was defined as having primary or higher education.

Table S4. Comparison of Baseline Characteristics between Ethnicities

Baseline characteristics	Malay (n=1526)	Indian (n=1837)	Chinese (n=2188)	P-value
Age, years	59.2 (11.0)	57.8 (10.1)	59.7 (9.9)	<0.001
Female Gender, n (%)	789 (51.7)	908 (49.4)	1104 (50.5)	0.422
Diabetes Mellitus, n (%)	423 (27.7)	651 (35.4)	334 (15.3)	<0.001
Hyperlipidaemia, n (%)	600 (39.8)	819 (46.0)	958 (44.9)	0.001
Hypertension, n (%)	953 (62.7)	1011 (55.1)	1210 (55.4)	<0.001
History of Cardiovascular Disease, n (%)	134 (8.8)	209 (11.4)	112 (5.1)	<0.001
Chronic Kidney Disease, n (%)	216 (14.7)	88 (4.9)	85 (4.0)	<0.001
Alcohol Intake, n (%)	28 (1.8)	240 (13.1)	250 (11.4)	<0.001
Current Smoking, n (%)	303 (19.9)	248 (13.5)	273 (12.5)	<0.001
Body Mass Index, kg/m^2	26.4 (5.1)	26.2 (4.8)	23.7 (3.7)	<0.001
Type of Housing, n (%)				
• 1-2 room public flat	178 (11.7)	54 (3.0)	23 (1.1)	<0.001
• 3-4 room public flat	1026 (67.4)	1066 (58.3)	1174 (53.8)	
• 5 room public flat and better	319 (20.9)	707 (38.7)	986 (45.2)	
Monthly Income, n (%)				
• <SGD $2,000	1267 (83.6)	1216 (68.0)	1328 (62.5)	<0.001
• ≥SGD $2,000	248 (16.4)	572 (32.0)	797 (37.5)	
Education Level, n (%)				
• No formal education	279 (18.3)	222 (12.1)	323 (14.8)	<0.001
• Formal education‡	1244 (81.7)	1609 (87.9)	1865 (85.2)	

SGD= Singapore Dollars

Data presented as mean (standard deviation) or number (percentage), where appropriate.

†P-value was estimated based on one-way analysis of variance or chi-squared test, where appropriate

‡Formal education was defined as having primary or higher education.

Table S6. 6-Year Incidence of Low Vision and Blindness (Based on WHO Definition, Better-Seeing Eye)

	Vision Status	Age (year)	Overall		Malay		Indian		Chinese	
			At risk	Case (%)	At risk	Case (%)	At risk	Case (%)	At risk	Case (%)
Based on Presenting Visual Acuity	Incident Low Vision*	40 - 49	1,739	31 (1.8)	506	7 (1.4)	645	16 (2.5)	588	8 (1.4)
		50 - 59	2,275	71 (3.1)	625	29 (4.6)	718	21 (2.9)	932	21 (2.3)
		60 - 69	1,532	83 (5.4)	382	38 (9.9)	514	19 (3.7)	636	26 (4.1)
		70+	680	72 (10.6)	197	40 (20.3)	178	8 (4.5)	305	24 (7.9)
		Total	6,226	257 (4.1)	1,710	114 (6.7)	2,055	64 (3.1)	2,461	79 (3.2)
		**Age-standardized† (95% CI)**	**4.26 (3.62 to 5.07)**		**8.04 (3.30 to 20.60)**		**3.15 (2.33 to 4.34)**		**3.46 (2.57 to 4.71)**	
	Incident Blindness*	40 - 49	1,810	0 (0.0)	536	0 (0.0)	666	0 (0.0)	608	0 (0.0)
		50 - 59	2,396	3 (0.1)	669	1 (0.1)	760	1 (0.1)	967	1 (0.1)
		60 - 69	1,684	2 (0.1)	420	1 (0.2)	562	0 (0.0)	702	1 (0.1)
		70+	848	10 (1.2)	263	5 (1.9)	207	1 (0.5)	378	4 (1.1)
		Total	6,738	15 (0.2)	1,888	7 (0.4)	2,195	2 (0.1)	2,655	6 (0.2)
		**Age-standardized† (95% CI)**	**0.35 (0.15 to 0.74)**		**1.35 (0.24 to 4.40)**		**0.18 (0.01 to 0.80)**		**0.17 (0.06 to 0.68)**	
Based on Best-Corrected Visual Acuity	Incident Low Vision*	40 - 49	1,806	5 (0.3)	533	3 (0.6)	665	1 (0.2)	608	1 (0.2)
		50 - 59	2,385	13 (0.5)	662	9 (1.4)	759	3 (0.4)	964	1 (0.1)
		60 - 69	1,665	22 (1.3)	411	13 (3.2)	557	6 (1.1)	697	3 (0.4)
		70+	806	44 (5.5)	243	31 (12.8)	200	5 (2.5)	363	8 (2.2)
		Total	6,662	84 (1.3)	1,849	56 (3.0)	2,181	15 (0.7)	2,632	13 (0.5)
		**Age-standardized† (95% CI)**	**1.18 (0.89 to 1.63)**		**2.15 (1.60 to 7.10)**		**0.66 (0.32 to 1.37)**		**0.52 (0.23 to 1.22)**	
	Incident Blindness*	40 - 49	1,810	0 (0.0)	536	0 (0.0)	666	0 (0.0)	608	0 (0.0)
		50 - 59	2,394	1 (0.0)	667	1 (0.1)	760	0 (0.0)	967	0 (0.0)
		60 - 69	1,685	1 (0.1)	419	0 (0.0)	563	0 (0.0)	703	1 (0.1)
		70+	850	6 (0.7)	263	2 (0.8)	208	0 (0.0)	379	4 (1.1)
		Total	6,739	8 (0.1)	1,885	3 (0.2)	2,197	0 (0.0)	2,657	5 (0.2)
		**Age-standardized† (95% CI)**	**0.15 (0.04 to 0.46)**		**0.82 (0.04 to 4.16)**		**0.00**		**0.14 (0.04 to 0.65)**	

CI= Confidence Interval

*Based on WHO definition – low vision was defined as <20/60 but ≥20/400; blindness was defined as <20/400.

†Incidence were evaluated and standardized to Singapore Population Census 2010.

Table S7. 6-Year Incidence of Low Vision and Blindness, Stratified by Sex (Based on Better-Seeing Eye; US and WHO Definition)

	Vision Status	Age (year)	US Definition*						WHO Definition**					
			Overall		Male		Female		Overall		Male		Female	
			At risk	Case (%)	At risk	Case (%)	At risk	Case (%)	At risk	Case (%)	n	Case (%)	n	Case (%)
Based on Presenting Visual Acuity	Incident Low Vision	40 - 49	1,657	85 (5.1)	778	38 (4.9)	879	47 (5.3)	1,739	31 (1.8)	803	13 (1.6)	936	18 (1.9)
		50 - 59	2,087	143 (6.9)	996	68 (6.8)	1091	75 (6.9)	2,275	71 (3.1)	1072	32 (3.0)	1203	39 (3.2)
		60 - 69	1,299	164 (12.6)	691	93 (13.5)	608	71 (11.7)	1,532	83 (5.4)	791	42 (5.3)	741	41 (5.5)
		70+	501	115 (23.0)	280	66 (23.6)	221	49 (22.2)	680	72 (10.6)	363	38 (10.5)	317	34 (10.7)
		Total	5,544	507 (9.2)	2745	265 (9.7)	2799	242 (8.6)	6,226	257 (4.1)	3029	125 (4.1)	3197	132 (4.1)
		**Age-standardized† (95% CI)**	**9.4 (8.5 to 10.3)**		**9.5 (8.4 - 10.8)**		**9.2 (8.0 - 10.5)**		**4.3 (3.6 to 5.1)**		**3.9 (3.2 - 4.7)**		**4.2 (3.5 - 5.0)**	
	Incident Blindness	40 - 49	1,805	0 (0.0)	835	0 (0.0)	970	0 (0.0)	1,810	0 (0.0)	835	0 (0.0)	975	0 (0.0)
		50 - 59	2,390	3 (0.1)	1113	1 (0.1)	1277	2 (0.2)	2,396	3 (0.1)	1114	2 (0.2)	1282	1 (0.1)
		60 - 69	1,680	5 (0.3)	853	3 (0.4)	827	2 (0.2)	1,684	2 (0.1)	854	0 (0.0)	830	2 (0.2)
		70+	840	15 (1.8)	434	6 (1.4)	406	9 (2.2)	848	10 (1.2)	435	2 (0.5)	413	8 (1.9)
		Total	6,715	23 (0.3)	3235	10 (0.3)	3480	13 (0.4)	6,738	15 (0.2)	3238	4 (0.1)	3500	11 (0.3)
		**Age-standardized† (95% CI)**	**0.3 (0.2 to 0.5)**		**0.3 (0.1 - 0.5)**		**0.4 (0.2 - 0.7)**		**0.4 (0.2 to 0.7)**		**0.1 (0.0 - 0.3)**		**0.3 (0.2 - 0.6)**	
Based on Best-Corrected Visual Acuity	Incident Low Vision	40 - 49	1,800	12 (0.7)	832	5 (0.6)	968	7 (0.7)	1,806	5 (0.3)	834	2 (0.2)	972	3 (0.3)
		50 - 59	2,370	34 (1.4)	1110	15 (1.4)	1260	19 (1.5)	2,385	13 (0.5)	1111	2 (0.2)	1274	11 (0.9)
		60 - 69	1,628	75 (4.6)	833	36 (4.3)	795	39 (4.9)	1,665	22 (1.3)	845	8 (0.9)	820	14 (1.7)
		70+	721	96 (13.3)	385	46 (11.9)	336	50 (14.9)	806	44 (5.5)	423	16 (3.8)	383	28 (7.3)
		Total	6,519	217 (3.3)	3160	102 (3.2)	3359	115 (3.4)	6,662	84 (1.3)	3213	28 (0.9)	3449	56 (1.6)
		**Age-standardized† (95% CI)**	**3.3 (2.9 to 3.8)**		**3.0 (2.4 - 3.7)**		**3.6 (2.9 - 4.3)**		**1.2 (0.9 to 1.6)**		**0.8 (0.5 - 1.2)**		**1.7 (1.2 - 2.2)**	
	Incident Blindness	40 - 49	1,809	0 (0.00)	835	0 (0.0)	974	0 (0.0)	1,810	0 (0.0)	835	0 (0.0)	975	0 (0.0)
		50 - 59	2,393	1 (0.04)	1113	0 (0.0)	1280	1 (0.1)	2,394	1 (0.0)	1114	0 (0.0)	1280	1 (0.1)
		60 - 69	1,683	2 (0.1)	855	2 (0.2)	828	0 (0.0)	1,685	1 (0.1)	856	0 (0.0)	829	1 (0.1)
		70+	847	11 (1.3)	435	2 (0.5)	412	9 (2.2)	850	6 (0.7)	435	1 (0.2)	415	5 (1.2)
		Total	6,732	14 (0.2)	3238	4 (0.1)	3494	10 (0.3)	6,739	8 (0.1)	3240	1 (0.0)	3499	7 (0.2)
		**Age-standardized† (95% CI)**	**0.2 (0.1 to 0.4)**		**0.1 (0.0 - 0.3)**		**0.3 (0.2 - 0.6)**		**0.2 (0.0 to 0.5)**		**0.0 (0.0 - 0.2)**		**0.2 (0.1 - 0.4)**	

CI= Confidence Interval

*Based on US definition – low vision was defined as <20/40 but ≥20/200; blindness was defined as <20/200.

**Based on WHO definition – low vision was defined as <20/60 but ≥20/400; blindness was defined as <20/400.

†Incidence were evaluated and standardized to Singapore Population Census 2010.

Table S9. Population Attributable Risk (PAR) of Factors Associated with Incident Presenting and Best-Corrected Visual Impairment

	Population Attributable Risk* (%; PAR)	
**Baseline characteristics**	**Incident Presenting VI** **	**Incident Best-Corrected VI** **
Age (>60 years)	27.1	60.4
Ethnicity		
• Malay	15.4	25.3
History of Cardiovascular Disease	4.21	6.11
Chronic Kidney Disease	-	14.3
Current Smoking	4.19	-
Type of Housing		
• Smaller housing type†	24.7	39.5
Monthly Income, n (%)		
• <SGD $2,000	26.4	-
Education Level, n (%)		
• No formal education	14.1	29.3

VI= Visual Impairment, SGD= Singapore Dollars

*PAR refers to reduction in disease incidence if the risk factor was eliminated

**Based on US definition – VI was defined as VA<20/40

†Smaller housing type consists of 1 to 4 room public flat

Table S11a. Primary Causes of Incident Presenting Visual Impairment over 6 years, Stratified by Ethnicity

Causes of VI*	Incident Presenting Low Vision* (n=507)			Incident Presenting Blindness* (n=23)		
	Malay (n=209)	Indian (n=131)	Chinese (n=167)	Malay (n=15)	Indian (n=3)	Chinese (n=5)
**Cataract**	89(42.6%)	42(32.1%)	65(38.9%)	13(86.7%)	2(66.7%)	4(80.0%)
**Under-Corrected Refractive Error**	101(48.3%)	67(51.1%)	81(48.5%)	1(6.7%)	0(0.0%)	0(0.0%)
**Glaucoma**	1(0.5%)	0(0.0%)	2(1.2%)	1(6.7%)	0(0.0%)	0(0.0%)
**Diabetic Retinopathy**	5(2.4%)	8(6.1%)	1(0.6%)	0(0.0%)	0(0.0%)	0(0.0%)
**Age-Related Macular Degeneration**	5(2.4%)	5(3.8%)	2(1.2%)	0(0.0%)	0(0.0%)	0(0.0%)
**Amblyopia**	0(0.0%)	2(1.5%)	0(0.0%)	0(0.0%)	0(0.0%)	0(0.0%)
**Corneal Diseases**	1(0.5%)	0(0.0%)	1(0.6%)	0(0.0%)	0(0.0%)	0(0.0%)
**Maculopathy**	0(0.0%)	1(0.8%)	2(1.2%)	0(0.0%)	0(0.0%)	0(0.0%)
**Epiretinal Membrane**	1(0.5%)	0(0.0%)	1(0.6%)	0(0.0%)	0(0.0%)	0(0.0%)
**Posterior Capsular Opacification**	1(0.5%)	5(3.8%)	7(4.2%)	0(0.0%)	1(33.3%)	0(0.0%)
**Pterygium**	1(0.5%)	0(0.0%)	0(0.0%)	0(0.0%)	0(0.0%)	1(20.0%)
**Retinal Vein Occlusion**	1(0.5%)	1(0.8%)	0(0.0%)	0(0.0%)	0(0.0%)	0(0.0%)
**Myopia Maculopathy**	2(1.0%)	0(0.0%)	3(1.8%)	0(0.0%)	0(0.0%)	0(0.0%)
**Others**	1(0.5%)	0(0.0%)	2(1.2%)	0(0.0%)	0(0.0%)	0(0.0%)

VI= Visual Impairment; N/A= Not Applicable

Data presented as number (percentage)

*Based on US definition – low vision was defined as <20/40 but ≥20/200; blindness was defined as <20/200

Table S11b. Primary Causes of Incident Best-Corrected Visual Impairment over 6 years, Stratified by Ethnicity

Causes of VI*	Incident Best-Corrected Low Vision* (n=217)			Incident Best-corrected Blindness* (n=14)		
	Malay (n=108)	Indian (n=47)	Chinese (n=62)	Malay (n=9)	Indian (n=2)	Chinese (n=3)
Cataract	80(74.1%)	30(63.8%)	45(72.6%)	7(77.8%)	0(0.0%)	2(66.7%)
Under-Corrected Refractive Error	0(0.0%)	0(0.0%)	0(0.0%)	0(0.0%)	0(0.0%)	0(0.0%)
Glaucoma	3(2.8%)	0(0.0%)	2(3.2%)	1(11.1%)	0(0.0%)	0(0.0%)
Diabetic Retinopathy	8(7.4%)	5(10.6%)	0(0.0%)	0(0.0%)	0(0.0%)	0(0.0%)
Age-Related Macular Degeneration	8(7.4%)	3(6.4%)	5(8.1%)	0(0.0%)	0(0.0%)	0(0.0%)
Amblyopia	1(0.9%)	1(2.1%)	0(0.0%)	0(0.0%)	0(0.0%)	0(0.0%)
Corneal Diseases	0(0.0%)	0(0.0%)	2(3.2%)	0(0.0%)	0(0.0%)	0(0.0%)
Maculopathy	3(2.8%)	2(4.3%)	3(4.8%)	0(0.0%)	0(0.0%)	0(0.0%)
Epiretinal Membrane	1(0.9%)	1(2.1%)	0(0.0%)	0(0.0%)	0(0.0%)	0(0.0%)
Posterior Capsular Opacification	2(1.9%)	5(10.6%)	1(1.6%)	0(0.0%)	2(100.0%)	0(0.0%)
Pterygium	0(0.0%)	0(0.0%)	0(0.0%)	0(0.0%)	0(0.0%)	1(33.3%)
Retinal Vein Occlusion	0(0.0%)	0(0.0%)	0(0.0%)	0(0.0%)	0(0.0%)	0(0.0%)
Myopia Maculopathy	1(0.9%)	0(0.0%)	1(1.6%)	0(0.0%)	0(0.0%)	0(0.0%)
Others	1(0.9%)	0(0.0%)	3(4.8%)	1(11.1%)	0(0.0%)	0(0.0%)

VI= Visual Impairment; N/A= Not Applicable

Data presented as number (percentage)

*Based on US definition – low vision was defined as <20/40 but ≥20/200; blindness was defined as <20/200

Supplementary Table S13. Association between Baseline Factors and Likelihood of Remaining Visually Impaired over 6 Years

	Subjects with Baseline VI* who Remained Visually Impaired over 6 Years			
	Presenting VI* (n= 438)		Best-Corrected VI* (n=94)	
**Baseline characteristics**	**Relative Risk** (95% CI)**	**P-value**	**Relative Risk** (95% CI)**	**P-value**
Age, per decade	1.17 (1.06 - 1.29)	0.001	1.22 (0.96 - 1.55)	0.101
Female Gender	1.02 (0.86 - 1.22)	0.794	1.10 (0.73 - 1.66)	0.633
Ethnicity				
• Chinese	Reference	-	Reference	-
• Malay	1.62 (1.35 - 1.94)	<0.001	1.56 (1.04 - 2.34)	0.030
• Indian	1.30 (1.06 - 1.58)	0.011	1.26 (0.82 - 1.94)	0.296
Diabetes Mellitus	1.03 (0.88 - 1.20)	0.718	1.03 (0.76 - 1.40)	0.840
Hyperlipidaemia	0.89 (0.77 - 1.03)	0.121	0.84 (0.62 - 1.15)	0.284
Hypertension	1.14 (0.93 - 1.38)	0.205	1.34 (0.73 - 2.44)	0.343
History of Cardiovascular Disease	0.98 (0.78 - 1.22)	0.839	1.54 (1.06 - 2.23)	0.022
Chronic Kidney Disease	1.04 (0.88 - 1.23)	0.626	0.75 (0.53 - 1.06)	0.104
Alcohol Intake	1.30 (0.94 - 1.80)	0.111	1.80 (0.81 - 4.01)	0.150
Current Smoking	0.90 (0.68 - 1.18)	0.443	0.76 (0.42 - 1.39)	0.375
Body Mass Index, kg/m^2	1.00 (0.98 - 1.01)	0.795	1.01 (0.97 - 1.04)	0.718
Type of Housing				
• 5 room public flat and better	Reference	-	Reference	-
• 1-2 room public flat	1.01 (0.79 - 1.29)	0.944	1.42 (0.86 - 2.35)	0.166
• 3-4 room public flat	0.85 (0.71 - 1.02)	0.083	0.97 (0.63 - 1.48)	0.881
Monthly Income, n (%)				
• ≥SGD $2,000	Reference	-	Reference	-
• <SGD $2,000	1.70 (1.16 - 2.50)	0.007	0.60 (0.16 - 2.23)	0.449
Education Level, n (%)				
• Formal education†	Reference	-	Reference	-
• No formal education	1.23 (1.04 - 1.45)	0.018	1.32 (0.91 - 1.91)	0.146

VI= Visual Impairment; VA= Visual Acuity; CI= Confidence Interval: SGD= Singapore dollars

*Based on US definition – visual impairment was defined as <20/40, based on better-seeing eye.

**Adjusted for age, gender, ethnicity, diabetes mellitus, hyperlipidaemia, hypertension, history of cardiovascular disease, chronic kidney disease, alcohol intake, current smoking, body mass index, type of housing, monthly income, and education level.

†Formal education was defined as having primary or higher education.

Table S14. Comparison of Baseline Characteristics between Included and Excluded Subjects

Baseline characteristics	Included* (n= 5,551)	Excluded** (n=2,491)	P-value†
Age, years	56.1 (8.9)	59.7 (11.1)	<0.001
Female Gender, n (%)	2801 (50.5)	1276 (51.2)	0.526
Ethnicity, n (%)			
• Malay	1526 (27.5)	964 (38.7)	<0.001
• Indian	1837 (33.1)	999 (40.1)	
• Chinese	2188 (39.4)	528 (21.2)	
Diabetes Mellitus, n (%)	1408 (25.4)	800 (32.1)	<0.001
Hyperlipidaemia, n (%)	2377 (43.8)	1065 (44.9)	0.403
Hypertension, n (%)	3174 (57.3)	1703 (68.6)	<0.001
History of Cardiovascular Disease, n (%)	455 (8.2)	294 (11.8)	<0.001
Chronic Kidney Disease, n (%)	389 (7.3)	349 (14.8)	<0.001
Alcohol Intake, n (%)	518 (9.3)	200 (8.1)	0.064
Current Smoking, n (%)	824 (14.9)	485 (19.5)	<0.001
Body Mass Index, kg/m^2	25.4 (4.4)	25.7 (5.1)	0.002
Type of Housing, n (%)			
• 1-2 room public flat	255 (4.6)	247 (10.0)	<0.001
• 3-4 room public flat	3266 (59.0)	1617 (65.1)	
• 5-room public flat and better	2012 (36.4)	618 (24.9)	
Monthly Income, n (%)			
• <SGD $2,000	3811 (70.2)	2043 (84.0)	<0.001
• ≥SGD $2,000	1617 (29.8)	390 (16.0)	
Education Level, n (%)			
• No formal education	824 (14.9)	683 (27.5)	<0.001
• Formal education‡	4718 (85.1)	1798 (72.5)	

SGD= Singapore Dollars

Data presented as mean (standard deviation) or number (percentage), where appropriate.

*Subjects included in the final analysis for incident presenting VI (based on US definition – VI was defined as VA<20/40; i.e. inclusive of low vision and blindness, based on better-seeing eye)

**Subjects excluded from the final analysis for incident presenting VI (not including death cases)

†P-value was estimated based on chi-squared or independent t-test, where appropriate

‡Formal education was defined as having primary or higher education.
